# Graphene oxide and indole-3-acetic acid cotreatment regulates the root growth of *Brassica napus* L. via multiple phytohormone pathways

**DOI:** 10.1186/s12870-020-2308-7

**Published:** 2020-03-06

**Authors:** Lingli Xie, Fan Chen, Hewei Du, Xuekun Zhang, Xingang Wang, Guoxin Yao, Benbo Xu

**Affiliations:** 1 Hubei Key Laboratory of Waterlogging Disaster and Agricultural Use of Wetland, College of Life Science, Yangtze University, Jingzhou, Hubei 434025 P.R. China; 2 Oil Crops Research Institute of the Chinese Academy of Agricultural Sciences, Wuhan, Hubei 430062 P.R. China; 3 Hubei Provincial Seed Management Bureau, Wuhan, Hubei 430070 P.R. China; 4 School of Life and Science Technology, Hubei Engineering University, Xiaogan, Hubei 432000 P.R. China

**Keywords:** Graphene oxide, Brassinolide, Gibberellin, Root growth, Transcript level

## Abstract

**Background:**

Studies have indicated that graphene oxide (GO) could regulated *Brassica napus* L. root growth via abscisic acid (ABA) and indole-3-acetic acid (IAA). To study the mechanism and interaction between GO and IAA further, *B. napus* L (Zhongshuang No. 9) seedlings were treated with GO and IAA accordance with a two factor completely randomized design.

**Results:**

GO and IAA cotreatment significantly regulated the root length, number of adventitious roots, and contents of IAA, cytokinin (CTK) and ABA. Treatment with 25 mg/L GO alone or IAA (> 0.5 mg/L) inhibited root development. IAA cotreatment enhanced the inhibitory role of GO, and the inhibition was strengthened with increased in IAA concentration. GO treatments caused oxidative stress in the plants. The ABA and CTK contents decreased; however, the IAA and gibberellin (GA) contents first increased but then decreased with increasing IAA concentration when IAA was combined with GO compared with GO alone. The *9-cis-epoxycarotenoid dioxygenase* (*NCED*) transcript level strongly increased when the plants were treated with GO. However, the *NCED* transcript level and ABA concentration gradually decreased with increasing IAA concentration under GO and IAA cotreatment. GO treatments decreased the transcript abundance of *steroid 5-alpha-reductase* (DET2) and *isochorismate synthase* 1 (ICS), which are associated with brassinolide (BR) and salicylic acid (SA) biosynthesis, but increased the transcript abundance of *brassinosteroid insensitive 1-associated receptor kinase* 1 (BAK1), *cam-binding protein 60-like G* (CBP60) and *calmodulin binding protein-like protein* 1, which are associated with BR and SA biosynthesis.

Last, GO treatment increased the transcript abundance of *1-aminocyclopropane-1-carboxylic acid synthase 2* (ACS2), which is associated with the ethylene (ETH) pathway.

**Conclusions:**

Treatment with 25 mg/L GO or IAA (> 0.5 mg/L) inhibited root development. However, IAA and GO cotreatment enhanced the inhibitory role of GO, and this inhibition was strengthened with increased IAA concentration. IAA is a key factor in the response of *B. napus* L to GO and the responses of *B. napus* to GO and IAA cotreatment involved in multiple pathways, including those involving ABA, IAA, GA, CTK, BR, SA. Specifically, GO and IAA cotreatment affected the GA content in the modulation of *B. napus* root growth.

## Background

Nanomaterials are defined as forms of material with at least one constituent dimension in the range of 1–100 nm. Carbon nanomaterials are types of engineered nanomaterials that are being increasingly utilized because of their excellent optical, catalytic, electrical, mechanical, and thermal properties [1]. By using carbon nanomaterials, researchers are currently resolving challenges in agriculture, such as plant disease, pesticide and stress [2]. GO is a kind of 2D nanomaterial and a functionalized form of graphene that has been increasingly applied in multiple domains since the invention of GO in 2004 [3].

Nanomaterials have been reported to improve the germination rate of rice seeds; increase the root growth of corn, tomato and cucumber; enhance the growth rate of coriander and garlic plants; protect the photosynthesis system; and aid in defense against plant disease [4, 5]. However, research has also indicated that nanomaterial treatments can result in decreased germination rates and photosynthetic efficiency, reduced root and shoot length, reduction of biomass, and reduced nutrient contents in soybean [6, 7]. The regulation of nanomaterials in plants is complex and dynamic and and depends on the type of nanoparticle, treatments (concentration, tduration and method), and phytohormone balance [8].

Although GO can regulate plant growth and development, its mechanism is not clear. Research has indicated that the response of plants to GO is closely related to the reactive oxygen species (ROS) pathway. ROS are normal products of plant cellular metabolism. However, stresses lead to excessive production of ROS, causing oxidative damage and cell death. The plant defense mechanism is activated in response to stress, and increased amounts of protective enzymes and antioxidants are synthesized, such as ascorbate peroxidase, catalase (CAT), and superoxide dismutase (SOD). Studies have shown that nanomaterials influence plant growth and development via the ROS pathway [9]. Research has shown that under stress conditions, plant growth and defense responses are regulated in a coordinated manner by the activity of several phytohormones, such as ABA, CTK, GA and IAA. In addition, studies have shown that nanomaterial treatments can alter the expression levels of genes involved in multiple pathways, including the stress responses, cell metabolism, electron transport, and ABA and IAA synthesis pathways [10].

Auxin involved in many aspects of plant growth and development in the form of IAA. This hormone is involved in regulating the growth of the main roots, lateral roots, adventitious roots, root hairs, and vascular tissue. Mostly, Low concentrations of exogenous auxin mostly promote root growth, while concentrations of exogenous auxin inhibit the expansion of the main roots and stimulate the development of lateral roots and adventitious roots. IAA is perceived by auxin receptors such as *TRANSPORT INHIBITOR RESPONSE 1* (TIR1) together with Aux/IAA proteins and auxin response factors (ARFs).

Our previous experiments have proven that GO treatment regulates the root growth of *Brassica napus* and that this root growth was significantly correlated with the IAA content [11]. To study the mechanism by which GO regulats plant root development and crosstalk between GO and IAA further, *B. napus* L seedlings (Zhongshuang No. 9) were treated with GO and IAA accordance with a two factor design, and the protective enzyme activity; hormone contents; and transcript levels of key genes involved in ABA, IAA, GA, CTK, BR, and SA were measured.

## Results

### Phenotype and phytohormone content of *B.napus* subjected to GO and IAA treatments

Nanomaterials are defined as material forms with at least one constituent dimension in the range of 1–100 nm, and GO is a kind of 2D nanomaterial that has been widely applied in biology, medicine, and chemistry, as well as in environmental protection.

Seedlings growth traits, specifically, root length, root fresh weight, stem length, number of lateral roots, and endogenous phytohormone content were measured on the 10th day after GO and IAA treatments. Analysis of variance revealed indicated that GO or IAA treatment significantly affected the growth of *B.napus* (root length, stem length, number of adventitious roots) and the GA, IAA, CTK and ABA contents in the seedlings. GO exhibited significant crosstalk with IAA to regulate *B.napus* growth (Table 1). Additional IAA treatments significantly influenced the root fresh weight. GO and IAA cotreatment significantly affected the root length; number of adventitious roots; and contents of IAA, CTK and ABA. However, the cotreatment did not significantly affect the stem length or root fresh weight (*P* < 0.05).

**Table 1:** Effects of GO and IAA treatments on the seedling growth and phytohormone content of *B. napus* on the 10th day after treatment

Variation source	Root length (cm)	Stem length (cm)	NO. of Adventitious roots	Root fresh weight (g)	ABA content (ng g ^−1^ FW)	IAA content (ng g ^− 1^ FW)	CTK content (ng g ^− 1^ FW)	GA content (mg g ^− 1^ FW)
GO	7.39^**^	1.13^**^	24.28^**^	0.054	161.51^**^	33.70^**^	156.95^**^	5723.51^**^
IAA	4.95^**^	1.19^**^	19.62^**^	0.045^**^	61.26^**^	67.17^**^	76.61^**^	8131.81^**^
GO*IAA	4.98^**^	1.09	14.14^**^	0.044	60.52^**^	40.43^**^	47.56^**^	7240.09^**^

“**“Indicates a significant effect, *P* < 0.01

**Fig. 1:**
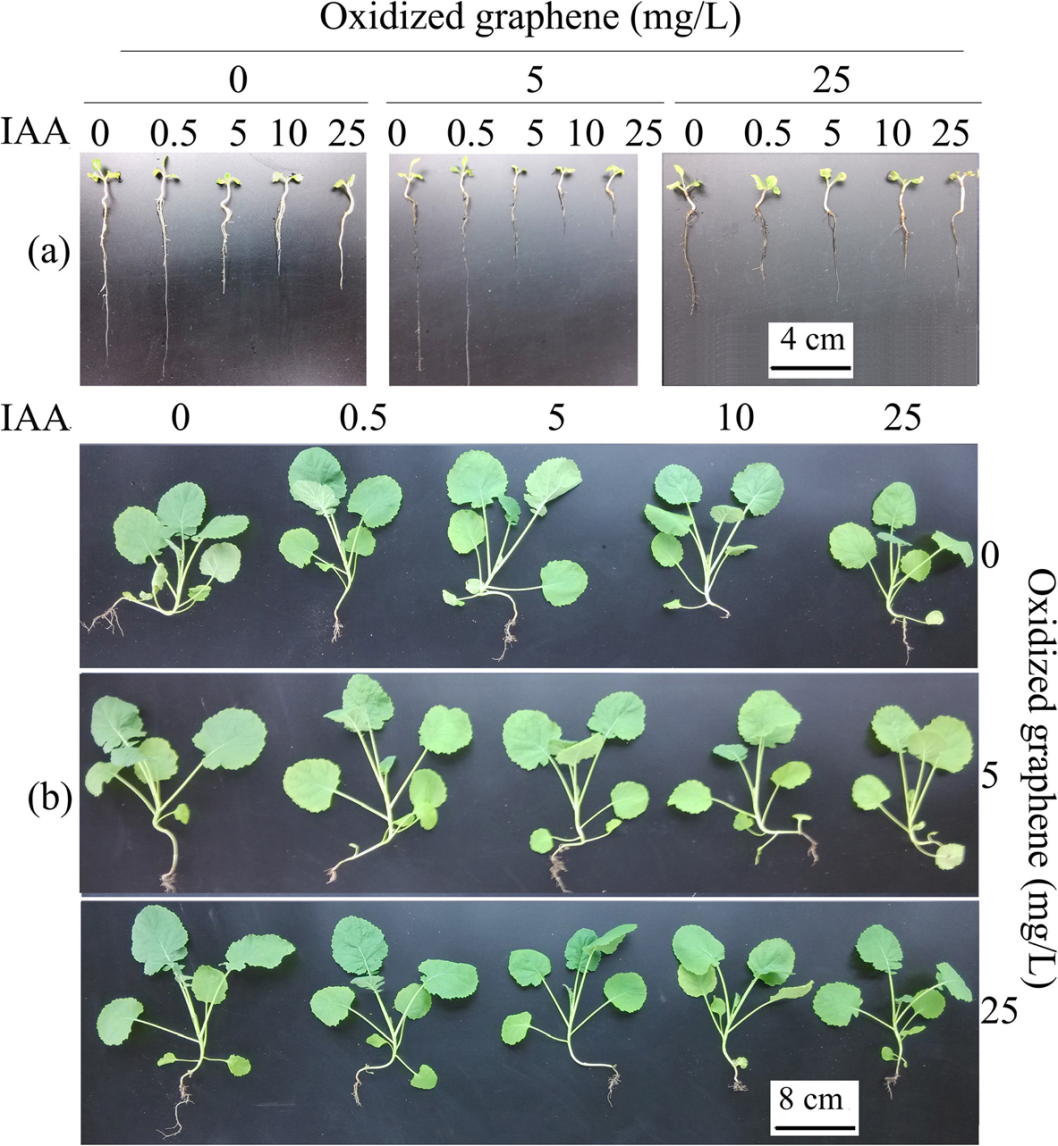
Phytohormone of *B.napus* seedlings on the 10th (**a**) and 30th (**b**) days after GO and IAA treatments

**Fig. 2:**
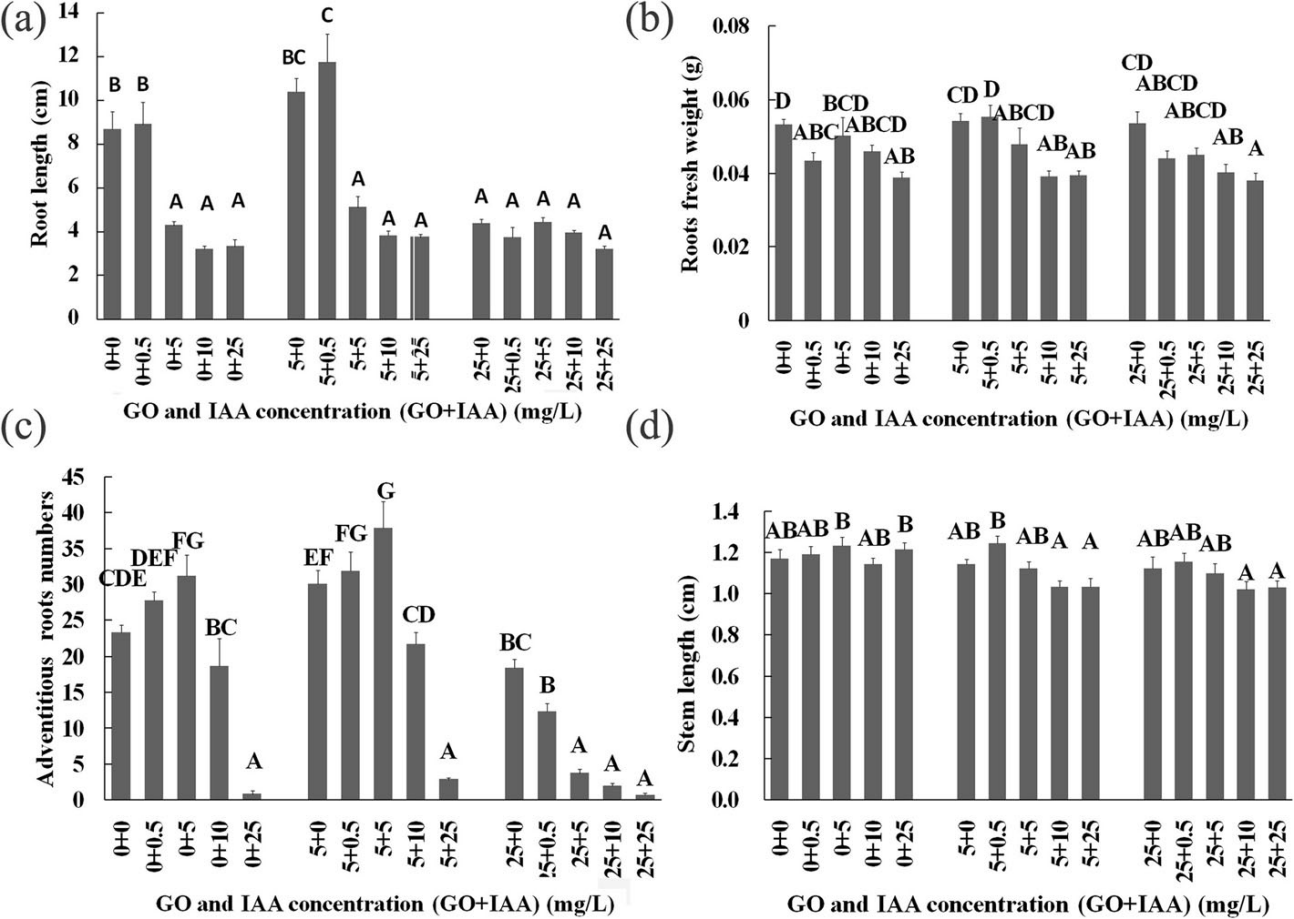
Root length (**a**), root fresh weight (**b**), number of adventitious roots (**c**) and stem length (**d**) of *B. napus* seedlings on the 10th day after GO and IAA treatments. The values with different letters are significantly different; Student’s t-test, *P* < 0.05 (lowercase letters) or *P* < 0.01 (uppercaseletters)

Compared with the control (CK) treatment (8.7 cm), the 5 mg/L GO treatment increased the root length (10.38 cm), but the 25 mg/L GO treatment suppressed root growth (4.39 cm) (Figs. 1 and 2a). The results showed that treatments with high concentrations of GO (> 25 mg/L) or IAA (> 0.5 mg/L) inhibited root development (Figs. 1a and 2a). Moreover, IAA cotreatment enhanced the role of the GO treatment, and the inhibition was strengthened with increasing IAA concentrations. The 0.5 mg/L IAA treatment did not significantly affect the root length, but the 5 mg/L GO cotreatment with 0.5 mg/L IAA promoted root growth, and the 0.5 mg/L IAA and 5 mg/L GO cotreatment significantly inhibited the root length, which further proved the crosstalk between GO and IAA (Fig. 2a). Parts of leaves cotreated with 25 mg/L GO and 10–25 mg/L IAA were necrotic. The results also proved that the 25 mg/L GO treatment was harmful to the seedlings and that IAA enhanced this disturbance.

The 5 and 25 mg/L GO treatments decreased the root fresh weight, and IAA cotreatment enhanced this effect. The 5 mg/L IAA treatment promoted adventitious root growth and increased number of adventitious root, but the 10–25 mg/L IAA treatments decreased the number of adventitious roots. Similarly, the 5 mg/L GO treatment increased the number of adventitious roots, whereas the 25 mg/L GO treatments decreased the number. The 5 mg/L GO and 0–5 mg/L IAA cotreatment increased the number of adventitious roots, but the 10–25 mg/L IAA and 5 mg/L GO cotreatment decreased the number of adventitious roots. Cotreatment with 25 mg/L GO and 0–25 mg/L IAA decreased the number of adventitious roots, and this repression was strengthened with increasing concentrations of IAA.

The IAA treatments did not affect the fresh weight or dry weight of the seedlings treated for 30 days, but the 25 mg/L GO treatment inhibited seedling growth (Fig. 2a). Cotreatment with 25 mg/L GO and 0–25 mg/L IAA inhibited the fresh weight and dry weight of seedlings treated for 30 days, and the inhibitory effect differed depending on the IAA concentration.

### Malondialdehyde (MDA) contents and root triphenyl tetrazolium chloride (TTC) activityare affected by GO and IAA treatment

**Fig. 3:**
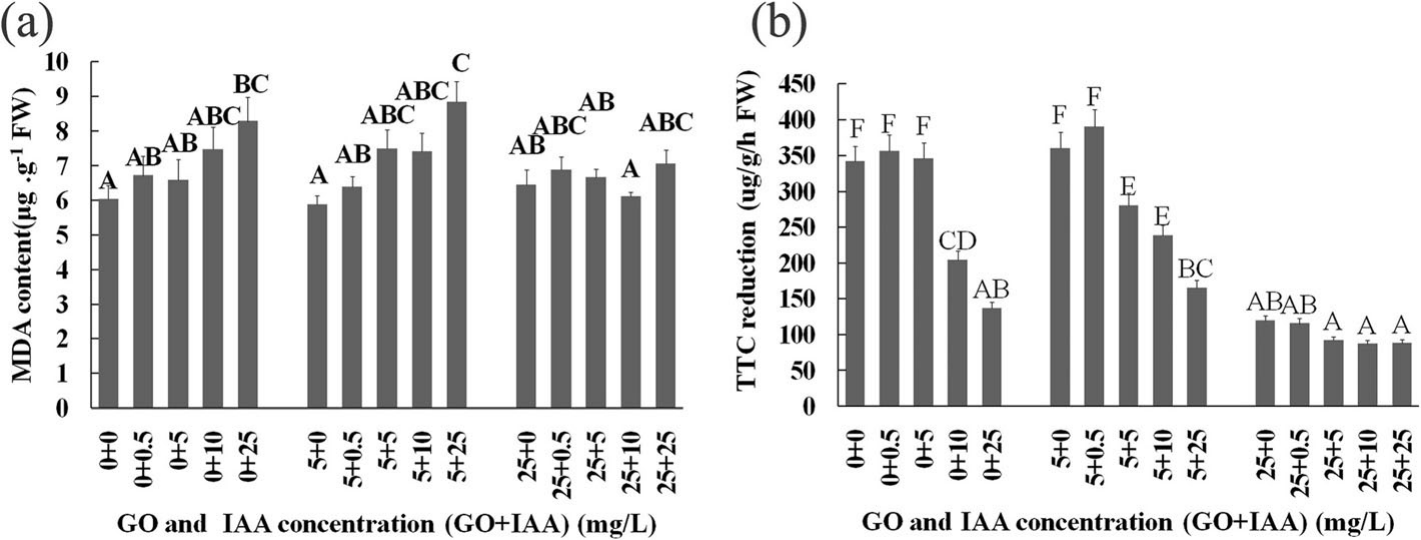
MDA content (**a**) and TTC reduction intensity (**b**) of seedlings on the 10th day after GO and IAA treatment. The values with different letters are significantly different; Student’s t-test, *P* < 0.05 (lowercase letters) or *P* < 0.01 (uppercaseletters)

IAA and GO cotreatment resulted in a high MDA content. In addition, 10–25 mg/L IAA or 25 mg/L GO treatments decreased the root TTC activity, but low-IAA and GO treatments had no significant inhibitory effect (Fig. 3).

### Phytohormone content s are affected by GO and IAA treatments

**Fig. 4:**
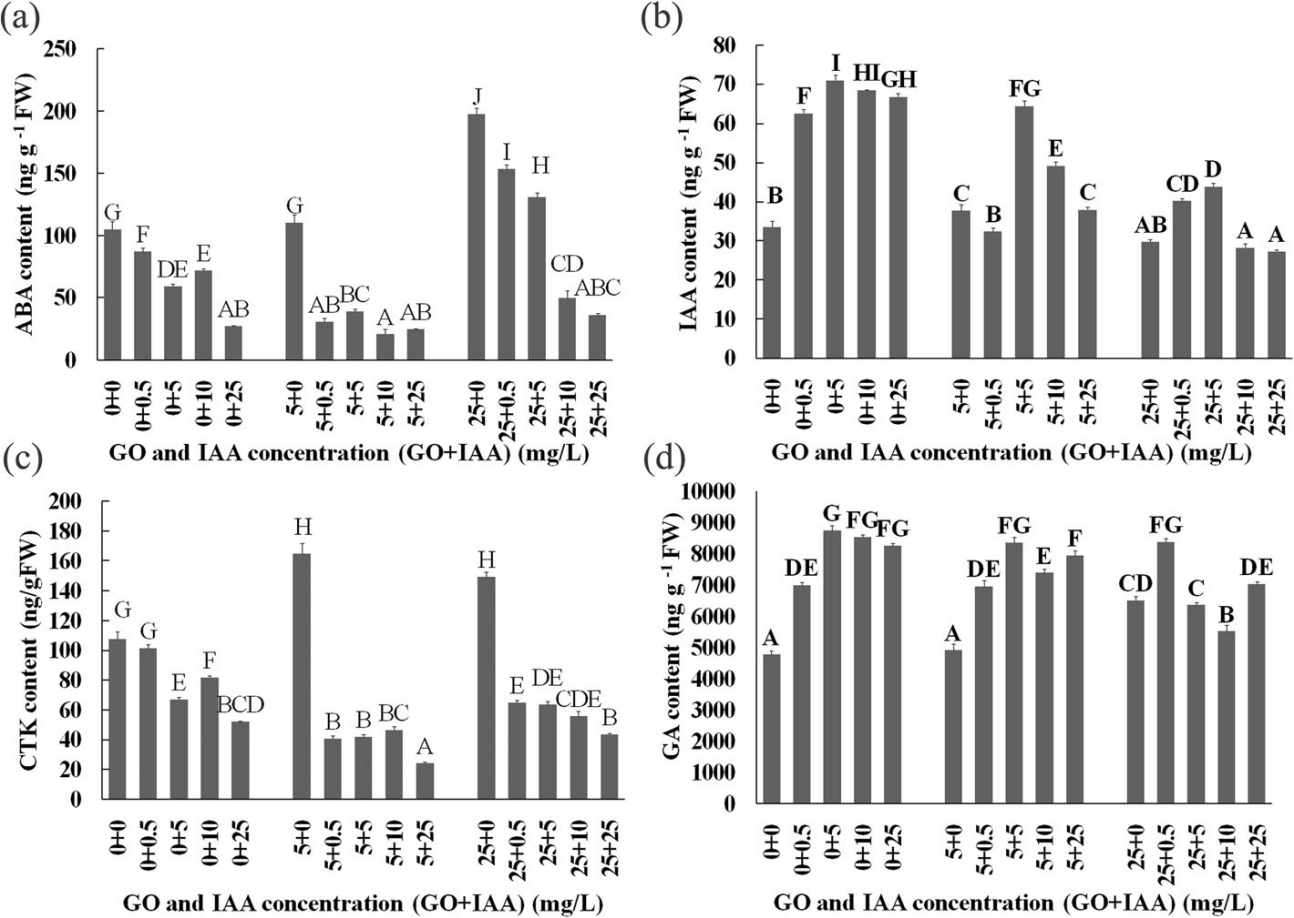
Contents of ABA(**a**), IAA(**b**), CTK(**c**) and GA (**d**) in *B.napus* seedlings on the 10th day after GO and IAA treatment. Values with different letters are significantly different (Student’s t-test, *P* < 0.01)

The results indicated that IAA treatment decreased the ABA and CTK contents but GO treatment increased the ABA and CTK contents. The ABA and CTK contents decreased with increasing IAA concentrations in response to the GO and IAA cotreatment compared with the GO treatment (Fig. 4a and c).

Generally, IAA contents increase with IAA increasing treatment concentrations, and our results showed a similar increase. The 5 mg/L GO treatment increased the IAA content, but the 25 mg/L GO treatment reduced the IAA content. Under the GO and IAA cotreatment, the endogenous IAA content first increased but then decreased with increasing IAA concentration from 0 to 25 mg/L (Fig. 4b).

The endogenous GA content first increased but then decreased with increasing IAA concentration. The 5 mg/L GO treatment did not alter the GA content, but the 25 mg/L GO treatment resulted in high GA content. Under GO and IAA cotreatment, the endogenous GA content also first increased but then decreased with increasing IAA concentration (Fig. 4d).

### Transcript levels of key genes involved in phytohormone pathways are affected by GO and IAA treatment

**Fig. 5:**
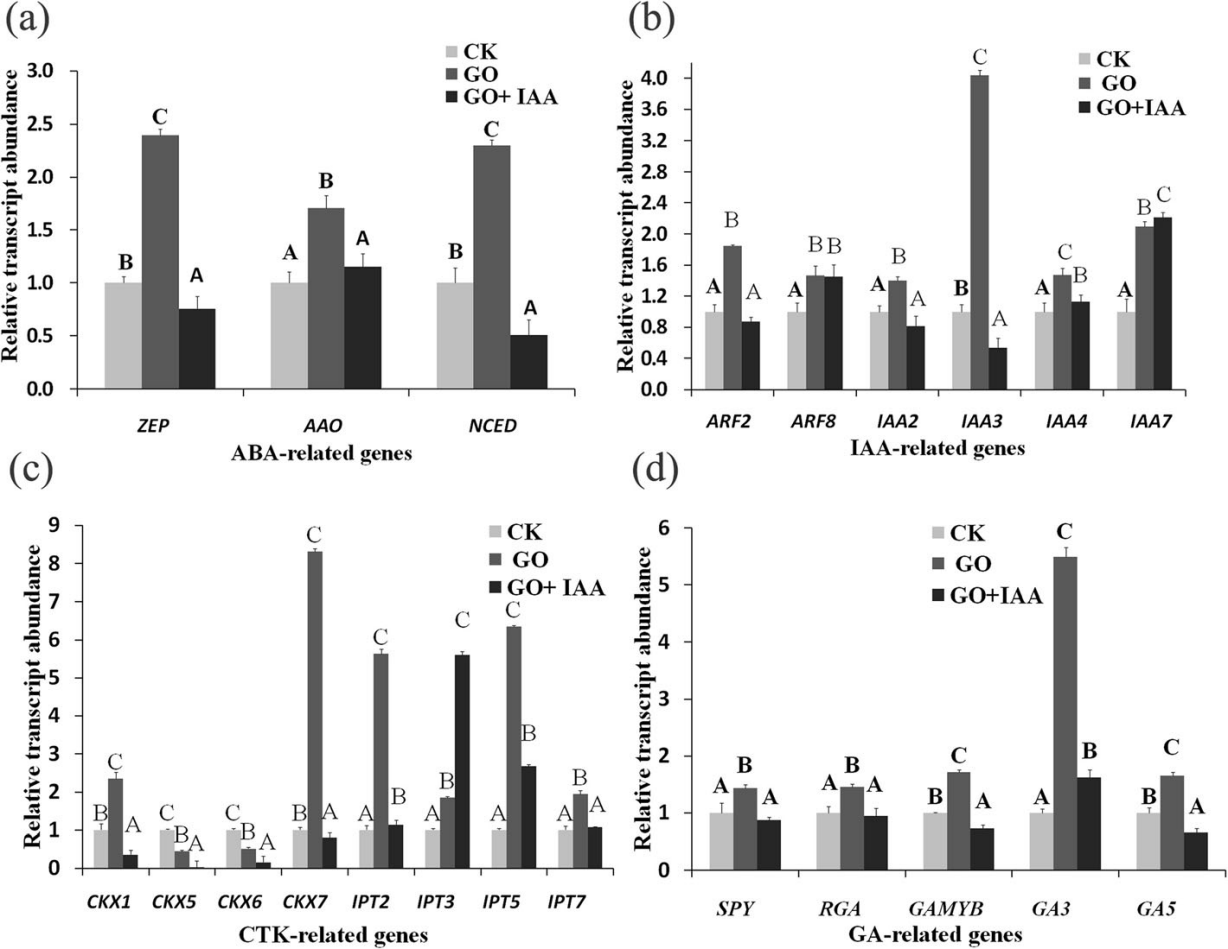
Relative transcript levels of key genes involved in the ABA (**a**), CTK (**b**) and GA (**c**) and IAA (**d**) pathways in *B. napus* treated with 25 mg/L GO and 10 mg/L IAA on the 10th day after treatment. Values with different letters are significantly different (Student’s t-test, *P* < 0.01)

Compared with the CK treatment, the 25 mg/L GO treatment increasedthe transcript levels of zeaxanthin epoxidase (*ZEP*), abscisic acid aldehyde oxidase (*AAO*) and *NCED*, but compared with the GO treatment, the 25 mg/L GO and 10 mg/L IAA cotreatment reduced the transcript abundance of these three genes, and the *ZEP* and *NCED* transcript levels were lower than those in the CK treatment (Fig. 5a).

The transcript levels of *ARF2*, *ARF8*, *IAA2*, *IAA3*, *IAA4* and *IAA7* increased under the 25 mg/L GO treatment. Compared with the GO treatment, the 25 mg/L GO and 10 mg/L IAA cotreatment reduced the transcript levels of *ARF2*, *IAA2*, and *IAA3* but increased the transcript level of *IAA7*; however, there were significant effects on the *ARF8* transcript level (Fig. 5b).

The 25 mg/L GO treatment increased the transcript levels of key genes involved in CTK and GA biosynthesis, but compared with the GO treatment, the 25 mg/L GO and 10 mg/L IAA cotreatment reduced the transcript abundance, except for that of *CKX5*, *CKX6* and *IPT3* (Fig. 5c and d).

**Fig. 6:**
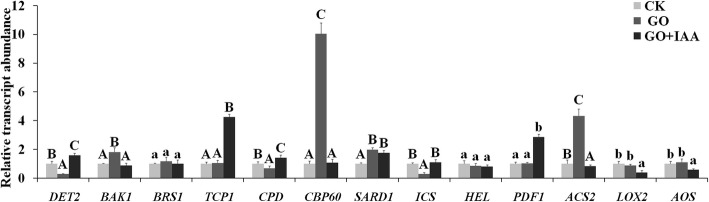
Relative transcript levels of key genes involved in the JA, BR, SA and ETH pathways in *B. napus* treated with 25 mg/L GO and 10 mg/L IAA on the 10th day after treatment. Values with different letters are significantly different (Student’s t-test, *P* < 0.01)

GO treatments decreased the transcript abundance of *DET2* and increased the transcript abundance of *BAK1*; however, GO treatment did not alter the transcript abundance of serine carboxy peptidase (*BRS1*) and *TCP1*, which are involved in BR biosynthesis (Fig. 6). Compared with the CK and GO treatments, GO and IAA cotreatment improved the transcript levels of *DET2* and *TCP1*, but compared with the GO treatment, the cotreatment inhibited the transcription of *BAK1*.

GO treatments resulted in increased transcription of *ICS* but decreased transcription of *CBP60* and systemic acquired resistance-deficient 1 (*SARD1*), which are key genes involved inthe SA pathway. Compared with the GO treatment, GO and IAA cotreatment inhibited *CBP60* transcription but had no significant effect on *SARD1* transcription.

GO treatment did not affect the transcript abundance of *LOX2* or allene oxide synthase (*AOS*), which are key genes involved in the jasmonic acid (JA) pathway, and had no significant effect on the transcript levels of Hevein-like protein (*HEL*) and *PDF1*, which are important genes for JA- and ETH-induced defense-related responses; however, GO treatment did increase the transcript levels of *ACS2* (a key gene involved in ETH biosynthesis)*.* Cotreatment with GO and IAA inhibited the transcription of *LOX2*, *AOS* and *ACS2*. By contrast, GO and IAA cotreatment improved the transcript abundance of the JA- and ETH- induced defense-related gene *PDF1*. Studies have shown that GO and IAA regulate plant growth via different pathways, but that crosstalk exists between GO and IAA.

Correlation analysis indicated that the root length was weakly correlated with the GA content (*r* = 0.26) but was not correlated with the ABA, IAA or CTK content, after GO and IAA cotreatment, which contrasted with our previous findings (GO modulation of rice root growth is dependent on the IAA content) [12]. Exogenous IAA can be applied, which could lead to a high IAA content in plants.

## Discussion

### Plant responses to nanomaterials depends on multiple factors

As an exogenously applied material with unique properties, GO can regulate the growth and development of plants either directly or indirectly. The accumulation of nanomaterials in plants has been shown to increase the shoot length, chlorophyll b content, number of adventitious roots, and fresh root weight of rice seedlings [13]. A 500 mg/kg CeO_2_ treatment was shown to increase the plant height, chlorophyll content, and biomass of barley without any toxic effects [14]. GO treatments decreased the damage caused by Cu stress by neutralizing the effects of Cu on nutrient accumulation in *Lemna minor* [3], and TiO_2_ nanoparticles have been reported to improve phosphorus uptake and improving plant growth [15]. GO treatments (25–100 mg/L) inhibited root growth and have negative effects on *B. napus* [8], and 2000 mg/L CeO_2_ inhibited the seed germination of corn, tomato and cucumber [16]. Our results indicated that GO or IAA treatment significantly affected the root length, stem length, and number of adventitious roots of *B. napus* seedlings. The 25 mg/L GO and 10 mg/L IAA cotreatment significantly inhibited the root growth, root fresh weight and number of adventitious roots, and inhibition was enhanced with increasing IAA concentration. The 25 mg/L GO treatment was harmful to the seedlings, which not only inhibiting root growth but also causing leaf necrosis. The effect of GO on plants depended on the concentration and treatment duration. The root length of five rice varieties treated with GO was correlated with the IAA content [11]. The research further proved that IAA had an important role in the response to GO in plants. Our results were consistent with the results in which low concentrations of GO increased plant root length, but in which high concentrations inhibited plant growth. Overall, the results indicated that the response of plants to nanomaterials depends on the plant genotype; content of endogenous phytohormone content; and the concentration, structure and localization of the nanomaterials within the plant [13, 17].

### The ROS pathway clearly regulates plant growth via GO despite the complexity of the mechanism involved

Nanomaterials cause an overproduction of ROS, subsequently resulting in oxidative stress, and lipid peroxidation, causing damage to plant proteins and DNA [18]. Studies have also demonstrated that nanoparticle treatments can improve the potential to scavenge ROS and increase antioxidant enzymatic activities to regulate growth processes in plants [17].

Silver nanoparticles lead to differential expression of *MSD1*, *CSD1* and *FSD* genes in rice seedlings, which is related to oxidative stress tolerance [19]. RNA-seq results indicated that hundreds of genes respond to nanoparticles, including the genes genes involved in photosynthesis-related metabolism, nitrogen metabolism, sucrose and starch metabolism and phytohormone signal transduction pathways, as well as genes involved with antioxidant enzymes [20].

**Fig. 7:**
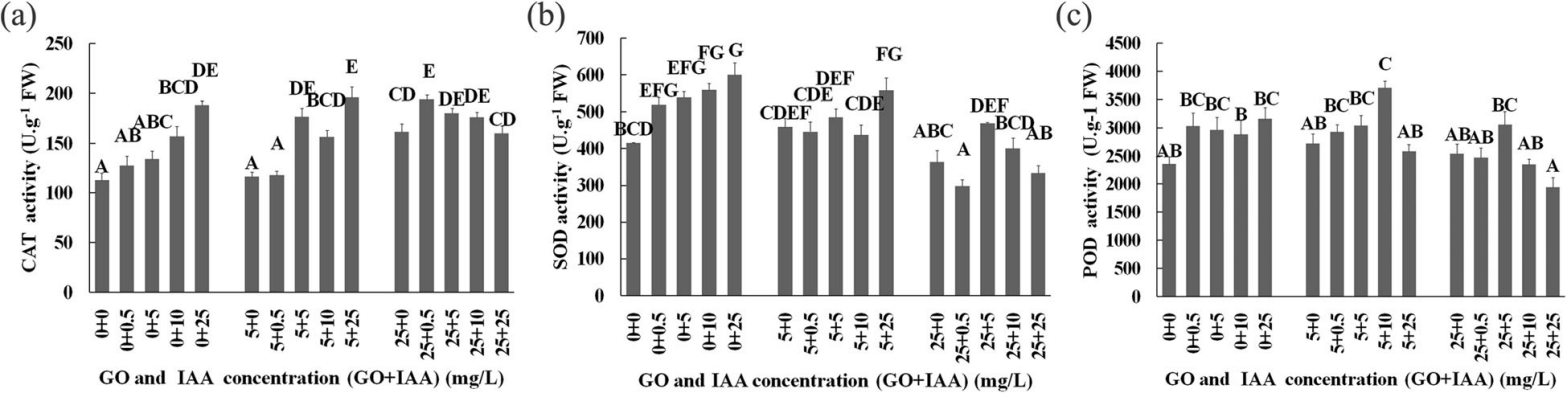
Activity of CAT (**a**), SOD(**b**) and POD (**c**) enzymes in seedlings on the 10th day after GO and IAA treatment. Values with different letters are significantly different (Student’s t-test, *P* < 0.01)

Our results showed that the high-concentration GO treatments resulted in a high MDA content and high CAT, SOD, and peroxidase (POD) activities (Fig. 7), and the 10–25 mg/L IAA and 25 mg/L GO treatments decreased the root TTC activity. The low-IAA and GO treatments had no significant inhibitory effect on root TTC activity, but the 5 mg/L IAA and 5 mg/L GO cotreatment inhibited the root TTC activity. Overall, our results proved that GO treatments regulated oxidative stress in plants, but the effect depended on the GO and IAA concentration and treatment duration, which further indicated that IAA is related to the effect of GO treatments on plant growth and development.

### GO modulates plant root growth via crosstalk between multiple phytohormones

Plant hormones are considered important molecular signals that not only regulate plant growth and development but also respond to stress to improve plant tolerance. ABA is considered the primary plant stress hormone, and its content increases quickly in response to stress to improve plant stress tolerance [21]. Overexpression of the *auxin response factor* 5 gene increases carotenoid contents and increases tolerance to salt and drought in *Arabidopsis* [22]. GA is applied mainly as a growth-promoting hormone on the basis of its role in the role of increasing cell division and elongation, recently, research has shown that GA can improve plant tolerance to abiotic stress. In addition, CTK plays important roles in regulating plant growth and development, such as inhibiting lateral root initiation and leaf senescence, and regulating cell division and phloem differentiation [23]. CTK also plays an important role in controlling cell division and the maintenance of stem cells via cooperation with auxin. Additionally, cis-zeatin level increased in tissues exposed to various stresses [24], and ETH is involved in regulating seedling morphology, leaf senescence, and biotic and abiotic stress tolerance [25]. SA and JA have also been reported to play a large role in the response to biotic stress [26].

ABA biosynthesis starts with the hydroxylation and epoxidation of the C40 carotenoid β-carotene to produce the all-trans-xanthophylls zeaxanthin and violaxanthin. Violaxanthin is subsequently converted into 9-cis-epoxyxanthophylls, and further converted into xanthoxin via the protein encoded by *NCED*. *NCED*, *AAO* and *ZEP* are 3 key genes involved in ABA biosynthesis. Auxin is perceived by auxin receptors, represented by *TIR1*, which results in the proteolysis of Aux/IAA proteins, thereby releasing their inhibitory effect on ARFs. IAA biosynthesis occurs via two pathways: tryptophan dependent and tryptophan independent pathways. ATP/ADP adenosine phosphate isopentenyl transferases (IPTs) are responsible for the synthesis of isopentenyladenine (iP)- and trans-zeatin (tZ)-type CTKs, while CTK degradation is catalyzed by cytokinin oxidase/dehydrogenase (CKX). GA derepresses the hormone response inhibited by DELLA proteins, including the *B. napus* DELLA protein (RGA) ga1–3, *RGL1*, *RGL2*, and *RGL3*.

A series of studies have shown that GO regulates hormone content in plants. GO treatment (50 mg/L) resulted in a relatively low IAA content and a relatively high ABA content because of high transcript levels of *NCED*, *AAO* and *ZEP* [8]. Cu nanoparticle treatments have been reported to activate defense mechanisms against stress and to increase the content of amino acids, ABA and phenolics [27]. Moreover, silver nanoparticle treatment increased cis-zeatin in pepper, which further proved that CTK is involved in stress responses in plants [2]. However, the mechanism of how hormones interact is not clear.

Several hormones, including ABA, BR and ETH, are important for regulating lateral root growth. ABA negatively regulates lateral root growth, and CTK-deficient CKX resulted in defects in lateral root spacing [28]. In addition, a relatively low CTK contentor signaling is always accompanied by a relatively high lateral root density [29]. CTK inhibits lateral root growth by blocking the cell cycle from the G2 stage to the M stage [30]. Auxin regulates multiple stages of lateral root growth, including the establishment of pericycle cells and the emergence of lateral roots [31]. However, CTK controls lateral root formation and growth by regulating the auxin gradient [32].

Auxin regulates CTK levels in the stem by inducing the expression of *CKX,* suppressing the expression of *IPT*, and promoting the expression of strigolactone biosynthesis-related genes [33]. CTKs modulate organogenesis by down regulating *PIN1* expression, and CYTOKININ RESPONSE FACTORS (CRFs) bind directly to the *PIN1* promoter to control *PIN1* expression in response to CTK [34].

Most importantly, GA treatment increases the number of primary roots. Studies have shown that overexpression of *GA2ox1* in *Populus* and overexpression of *RGL1* (resulting in GA-insensitive mutants) increased lateral root density and elongation [34] In addition, ETH affects lateral roots depending on the concentration: low ETH concentrations promote lateral root initiation, while higher concentrations doses inhibit lateral root initiation. The effect of BR on root elongation depends on the BR concentration; an appropriate concentration of BR promotes cell elongation, but a high concentration inhibits root growth. Moreover, compared with wild-type plants, *DET2* mutants display shorter roots [35].

Researchers have shown that ABA and auxin synergistically regulate plant growth. Exogenous ABA treatments have been reported to inhibit lateral root development. However, ABA is important for primary root elongation according to studies on ABA-deficient plants [36]. IAAs inhibit auxin signaling, while ARFs positively regulate the expression levels of auxin-induced genes [37]. Generally, ABA treatment represses *IAA7* expression but increases *ARF2* expression [38].

The product of the *NCED* gene is the rate-limiting step in the ABA biosynthesis pathway. Our results proved that the *NCED* transcript level strongly increased when plants were treated with GO, which resulted in a high ABA content and decreased root length, further proving that ABA negatively regulates lateral root growth. A previous report also proved that GO treatment resulted in increased ABA contents and decreased IAA contents [10]. However, under increasing IAA concentrations, GO and IAA cotreatment gradually decreased *NCED* transcript levels and ABA concentrations. In addition, GO treatment increased the length of seminal roots of the wild-type tomato but decreased length of seminal roots of transgenic plants (overexpressing *NCED*) [39]. ABA may be the primary hormone that responds to GO. Our results also indicated that IAA treatments increased the IAA content, but that GO treatments decreased the IAA content.

There are 23 ARF proteins in *Arabidopsis* that bind specifically to the auxin-responsive element (AUXRE) TGTCTC to regulate the transcription of auxin-responsive genes. IAAs inhibit auxin signaling, while ARFs promote the transcription of auxin-induced genes [37]. Auxin signaling via *ARF8* is essential for JA production [40]. *ARF2* inhibits transcription of *HOMEOBOX PROTEIN* 33 to regulate the repressive role of ABA in primary root growth [38]. The results of that study further indicated that IAA treatments increased the *ARF2* transcript level, but that IAA and GO cotreatment resulted in low *ARF2* transcript levels. The results of our experiment also showed that the 10 mg/L ABA treatment increased the *ARF2* transcript level but reduced the IAA content. Thirty-four dysregulated long noncoding RNAs, especially lnc37 and lnc14, were considered to be involved in the response to GO on the basis of genome-wide identification and functional analyses [41]. GO treatments significantly decreased the transcript levels of the auxin efflux carriers, *PIN7* and *ABCB1*, and of *ARR3* (a CTK response regulator) with increasing GO concentration. The low-concentration (1 mg/L) GO treatments increased the transcript levels of *ARRO1* and *TTG1*, but the high-concentration (10 mg/L) GO treatments inhibited the transcription of these genes, which are involved in root growth [10]. It is possible that the GO treatment increased the ABA content but then decreased the IAA content under high ABA concentration.

Auxin regulates CTK levels in the stem by inducing the expression of *CKX,* suppressing the expression of *IPT*, and promoting the expression of strigolactone biosynthesis-related genes [33]. Both ABA and auxin inhibit root growth by causing excess production of ROS.

Numerous studies have shown that stress results in a low CTK content. Studies have also shown that stress causes high CTK levels because multiple factors influence stress signaling. According to transcriptome and MapMan analyses, genes that respond to CTK are involved mainly in the response to abiotic stress [42]. CTK-deficient plants have reduced levels of ABA because of low CTK levels [43]. CTK enhances cotyledon greening by promoting the proteasomal degradation of *ABI5*, which induces the expression of *ARR5*, which is involved in lateral root formation [44]. CTK can also inhibit stomatal closure via direct interaction with NO, which is an important signaling molecule that plays a role in the ABA-mediated stomatal closure pathway [45]. These results indicate that ABA and auxin can regulate the CTK content.

We assume that GO treatments increased the ABA content but then decreased the IAA content as a result of the high ABA concentration. Furthermore, the low IAA content inhibited CKX transcription and resulted in a relatively low CTK content. However, this hypothesis needs further confirmation.

BR binds to BR-insensitive 1 (BRI1) and results in a rapid association between BRI1 and its coreceptor BRI1-associated receptor kinase 1 (BAK1)*.* BAK1 is involved in multiple signaling pathways and integrates several cell responses to regulate plant growth [46]. *BRS1* is a serine carboxy peptidase that was recognized to regulate cell elongation and shape formation, both of which govern the length of hypocotyls and secondary inflorescence branches [47]. Constitutive photomorphogenesis and dwarfism (CPD) encodes an ɑ-hydroxylase that participates in a key ɑ-hydroxylationstep in BR biosynthesis [48]. TCP1 encodes a TCP transcription factor that promotes DWF4 expression for BR biosynthesis [49]. The plant lipoxygenase (LOX) enzyme catalyzes the oxidation of polyunsaturated fatty acids, after which AOS catalyzes the transformation of hydroperoxy fatty acid to SA [50]. SARD1 and CBP60 can bind to the promoter of ICS1 and positively regulate the expression of *ICS1*, which encodes a key enzyme involved in pathogen-induced SA synthesis [51]. Allene oxide cyclase (AOX) catalyzes the conversion of epoxyoctadecatrienoic acid (OPDA) to JA via several enzymatic reaction steps [52]. ETH levels increase under excess metal concentrations. The expression of 1-aminocyclopropane-1-carboxylic acid synthase (ACS) and the accumulation of ETH are induced by Cd in *Arabidopsis thaliana* plants, mainly via the ACS pathway [47].

The transcript abundance of *DET2* and *ICS* decreased under GO treatments; these are key genes involved in the BR and SA pathways. By contrast, GO treatment increased the transcript abundance of *BAK1*, which is a key gene involved in BR biosynthesis, and *CBP60* and *SARD1*, which are important genes involved in the SA pathway. GO treatment also increased the transcript abundance of *ACS2* (involved in the ETH pathway) but had no significant effect on that of *LOX2* and *AOS* (involved in the JA pathway) or on *HEL* and *PDF1* (involved in the JA and ETH pathways). These results indicated that the response pathways also included those of BR, SA and ETH.

## Conclusions

In this study, *B.napus* seedlings were treated with GO and IAA, and the morphological characteristics and phytohormone contents of the treated seedlings were measured. GO and IAA significantly affected the root length; number of adventitious roots; and contents of IAA, CTK and ABA. IAA is an important phytohormone that regulates the root growth of *B. napus* L*.* under GO treatments, and the responses of *B. napus* to GO and IAA cotreatment involve multiple pathways, including the ABA, IAA, GA, CTK, BR, and SA pathways. Last, GO and IAA cotreatment affected the GA content in the modulation of *B. napus* root growth.

## Methods

### Plant growth and treatments

Zhongshuang No. 9 seeds were used as experimental materials and were provided Yong Chen (Oil Crops Research Institute of the Chinese Academy of Agricultural Sciences). The seeds were germinated in the dark in a growth chamber that had a 24-h photoperiodand a temperature of 25 ± 1 °C. GO was obtained from Suzhou Carbon Science and Technology [12].

Zhongshuang No. 9 seedlings (4 days old) that displayed identical growth were selected and cotreated with GO (0, 5, and 25 mg/L) and IAA (0, 0.5, 5, 10, and 25 mg/L) in accordance with a completely randomized two-factor design reported previously [53]. More than five of the seedlings cotreated with GO and IAA for 10 days were randomly selected to measure the root length, root fresh weight, and stem height according to the previously reported methods [8].

### Measurement of enzyme activities and MDA and phytohormone contents

The activity of POD, CAT and SOD enzymes was measured according to guaiacol oxidation method [54], H_2_O_2_ method [55] and nitro blue tetrazolium (NBT) method [15], respectively. The 2- thiobarbituric acid method [55] and TTC method [12] were used to measure the MDA content and root activity respectively. Phytohormones were extracted, purified and measured according to previously the reported methods [53, 56].

### Determination of transcript abundance

Total RNA was extracted and reverse transcribed into cDNA for qPCR, and the relative transcript level was calculated according to previously reported method [57] in conjunction with qPCR primers [53]. DPS 7.05 software was used for analysis of variance on the basis of significance at *P* < 0.05 (indicated by lowercase letters in this study) or *P* < 0.01 (indicated by uppercase letters in this study) [58].

## Data Availability

The data sets supporting the results of this article are included within the article.

## Abbreviations

AAO: Abscisic acid aldehyde oxidase
ABA: Abscisic acid
ACS: Aminocyclopropane-1-carboxylate synthase
ACS2: 1-aminocyclopropane-1-carboxylic acid synthase 2
AOS: Allene oxide synthase
ARF: Auxin response factor
BAK1: Brassinosteroid insensitive 1-associatedreceptor kinase 1
BRS1: Serine carboxypeptidase
CBP60: Cam-binding protein 60-like G
CKX: Cytokinin oxidase/dehydrogenase
CPD: Constitutive photomorphogenesis and dwarfism
CTK: Cytokinin
DET2: Steroid 5-alpha-reductase
ETH: Ethylene
GA: Gibberellin
GAMYB: Transcription factor MYB65
HEL: Hevein-like protein
IAA: Indole-3-acetic acid
ICS1: Isochorismate synthase 1
IPT: Adenosine phosphate isopentenyl transferase
JA: Jasmonate acid
LOX: Lipoxygenase
MDA: Malondialdehyde
NCED: 9-cis-epoxycarotenoid dioxygenase
RGA: *Brassica napus* DELLA protein
SA: Salicylic acid
SARD1: Systemic acquired resistance-deficient 1
SPY: UDP-N-acetylglucosamine-peptide N-acetylglucosaminyltransferase
TTC: Triphenyl tetrazolium chloride
ZEP: Zeaxanthin epoxidase
